# Effects of Drought-Stress on *Fusarium* Crown Rot Development in Barley

**DOI:** 10.1371/journal.pone.0167304

**Published:** 2016-12-09

**Authors:** Xinlun Liu, Chunji Liu

**Affiliations:** 1 College of Agronomy, State Key Laboratory of Crop Stress Biology for Arid Areas, Northwest A&F University, Yangling, Shaanxi, China; 2 CSIRO Agriculture & Food, St Lucia, Qld, Australia; 3 School of Plant Biology, The University of Western Australia, Perth, WA, Australia; University of Tasmania, AUSTRALIA

## Abstract

*Fusarium* crown rot (FCR), caused by various *Fusarium* species, is a chronic disease of cereals in many semi-arid regions worldwide. To clarify what effects drought-stress may have on FCR development, visual assessment, histological analysis and quantitative PCR were used to analyse the infection process of *F*. *pseudograminearum* in barley. This study observed for the first time that the severity of FCR symptom reflects the quantity of pathogens in infected tissues of barley under both drought-stressed and well-watered conditions. Drought-stress prolongs the initial infection phase but enhances the proliferation and spread of *Fusarium* pathogens after the initial infection phase. Under drought-stressed conditions, the invading hyphae were frequently observed to re-emerge from stomata and invade again the surrounding epidermis cells. Under the well-watered conditions, however, very few hyphae re-emerged from stomata and most infection was caused by hyphae intracellularly grown. It was also observed that drought-stress increased the length and density of trichomes dramatically especially in the susceptible genotypes, and that the length and density of trichomes were positively related to fungal biomass of *F*. *pseudograminearum* in plants.

## Introduction

*Fusarium* crown rot (FCR), primarily caused by *Fusarium pseudograminearum*, is a serious cereal disease in many arid regions worldwide [1, 2]. In Australia, FCR is one of the most economically significant diseases for wheat and barley [3, 4]. Compared to bread wheat, barley is more susceptible to FCR infection when assessed by stem browning but suffers less yield loss [5, 6]. In recent years, FCR incidence has dramatically increased due likely to increased intensity of cereal in cropping system and the wider adoption of minimum tillage for moisture conservation [7].

Infective propagules of *Fusarium* spp., as mycelia on residual stubble or durable chlamydospores in the soil, infect the emerging shoot of plants. They colonize the crown and stem base as they develop, and sequentially penetrate the leaf sheaths at the stem base [8, 9]. Symptoms associated with FCR include stand reductions, brown necrotic lesions on the coleoptile, roots, and subcrown internode, and the rotting of root, crown, and stem tissues [8]. Stem-base browning has been widely used to measure FCR resistance [9–11]. Previous data showed that the expression of FCR severity in the field is strongly dependent on level of rainfall and the degree of moisture stress late in the growing season [12]. Severe FCR often happens with a ‘wet start’ (good moisture during seedling stages) and ‘dry finish’ (water deficit toward the end of plant growth) [2, 8, 13, 14].

Despite of the widely held believe that drought-stress enhances FCR development, there is no report on whether the increased disease severity observed under such environments is due to changes in plant sensitivity to disease infection or changes in pathogen quantities accumulated in infected tissues. To further improve our understanding of drought-stress in plant-FCR interaction, we compared the progresses of FCR infection between drought-stressed and well-watered conditions based on visual assessment of disease severity, quantitative PCR to estimate pathogen quantity and histological analysis of infected tissues. Results obtained from these assessments and their possible implications are described in this publication.

## Materials and Methods

### Inoculum preparation

*F*. *pseudograminearum* isolate (CS3096), a highly aggressive isolate maintained in the CSIRO collection [15], was used in this study. Inoculum was prepared based on the method described by Li et al. [16]. The fungal spores were harvested and the concentration of the spore suspension was adjusted to 1x10^6^ spores mL^-1^, and Tween 20 was added to the spore suspension to a final concentration of 0.1% (v/v).

### Plant growth, inoculation procedure and disease assessment

Four barley genotypes with different levels of resistance to FCR were used. Two of them (CSCRB8003 and CSCRB8012) are resistant genotypes and the other two are susceptible commercial cultivars (Franklin and Fleet). Seeds were germinated in Petri dishes on three layers of filter paper saturated with water. The germinated seedlings were inoculated based on the method reported by Li et al. [16]. Inoculated seedlings and non-inoculated controls were planted in 3.5 cm square punnets filled with the University of California potting mix C (consisting of 50% sand and 50% peat v/v).

Two experiments were conducted, each consisting of three replicates (each replicate contains four groups, each group including 50 seedlings) in the controlled greenhouse at the CSIRO Brisbane laboratories. Plants in two of the four groups were inoculated and the other two were used as non-inoculated controls. Together with the non-inoculated controls, one of the inoculated groups had been well-watered (relative soil-water content was maintained between 100% and 80%) after inoculation and the others watered only when wilting appeared (at about 45% of relative soil-water content). Settings for the controlled greenhouse were as follow: 14 h day/ 10 h night cycle, 25 (±1)°C day/ 18 (±1)°C night temperature, and 65 (±5)% day/80 (±5)% night relative humidity.

FCR severity was evaluated for each of the three replicates. Samples were taken at 1, 3, 5, 7, 10, 14, 21 and 28 days post inoculation (dpi) and FCR severity was scored on a scale of 0 to 5 according to Li et al. [16]. A disease index (DI) was then calculated for each treatment following the formula of DI = ∑(Rn X ⁄ N), where X represents the scale value of each seedling, n represents the number of seedlings in the category, and N represents the total number of seedlings evaluated for each treatment.

### DNA extraction and fungal biomass estimation

Samples were collected from 5 plants for each genotype in each replicate at 1, 3, 5, 7, 10, 14, 21 and 28 dpi, respectively, by cutting the shoots between 0 and 2 cm above the soil surface with scissors. Fresh samples were frozen immediately in liquid nitrogen and then stored in a -80°C freezer. Frozen samples were ground by a Retsch MM300 Ball mill (Retsch GmbH, Haan, Germany) for DNA extraction. DNA was extracted using the QIAGEN DNeasy plant mini kit (Qiagen, Hilden, Germany). DNA was eluted into 100 μL of sterile water and stored at -20°C until used.

Fungal biomass was estimated by qPCR based on the proportion of fungal DNA to barley DNA. The barley *Actin* gene (AY145451) was used as the reference (forward primer 5’-GAACAGGAGCTGGAGACTGC-3’ and reverse primer 5’-ATCATGGATGGCTGGAAGAG-3’) and the fungal biomass in samples was estimated using the *Tri5* gene of *Fusarium* species (forward primer 5’-GCGCATCGAGAATTTGCA-3’ and reverse primer 5’-TGGCGAGGCTGAGCAAAG-3’). These genes have been previously used in estimating *Fusarium* biomass in barley tissues [6, 17]. The qPCR analysis was performed as described by Bai et al. [17]. *Fusarium* DNA relative to barley DNA was calculated using the following equation [18]:

$$Relative\;biomass=Ef^{-Ct}{}_{Fungal}/Ef^{-Ct}{}_{plant}$$

Where Ef represents PCR amplification efficiency determined using program LINREGPCR 7.5 [19] and Ct represents the crossing threshold. Logarithmic transformations were carried out on the original biomass data before used for further analysis.

### Tissue preparation and histological analysis

Coleoptile, the first leaf sheath (L1) and the second leaf sheath (L2) between 0 and 2 cm above the soil surface were used for histological analyses. Samples were cleaned and stained based on methods described by Schäfer et al. [20]. Briefly, tissues were placed in a clearing solution (4:1 ethanol with 0.15% trichloroacetic acid, chloroform) for 48 h. The samples were then washed 2 × 15 min with 50% ethanol, 2 × 15 min with 50 mM NaOH and 3 × 10 min with milli-Q water, and incubated for 30 min in 0.1 M Tris-HCl (pH 8.5). The samples were then stained for 10 min in 0.1% Fluorescent brightener 28 [with 0.1 M Tris-HCl (pH 8.5)] and finally washed 3 × 10 min with water. Samples were stored in 50% (v/v) glycerol. Stained samples were then examined using a ZEISS AX10 Fluorescence Microscope (http://microscopy.zeiss.com/microscopy/fr_fr/).

The length and number of trichomes were measured from the first leaf sheath of 14 dpi seedlings with the non-inoculated ones as controls. Five plants in each replicate were used and three microscopic fields per sheath were examined and their averages were used for statistical analysis.

### Statistical analyses

SPSS statistics 16.0 was used in analyzing the data obtained in this study. Statistical characteristics of continuous data are expressed as means and their standard deviations (SDs) and compared with one-way ANOVA and Student-Newman-Keuls test. The Student’s *t*-test for independent samples was used to compare the results of treatments. The Pearson correlation was estimated between fungal biomass, FCR severity and trichome characteristics. The threshold of statistical significance for all the comparative tests was set at p < 0.05.

## Results

### Effects of water deficit on FCR development

The initial symptom of FCR, represented by a few minute lesions at the base of coleoptiles, was detected from the two susceptible genotypes by 5 dpi. At 7 dpi, the typical symptom of FCR was detected in the coleoptiles of all four genotypes assessed. The mean disease ratings of the well-watered seedlings were higher than that of the drought-stressed ones at this initial infection stage (Table 1 and S1 Table).

**Table 1:** *Fusarium* crown rot disease index (DI) of the four barley genotypes assessed at 6 different time points following inoculation ^a^.

Barley genotype	Treatment	5 dpi	7 dpi	10 dpi	14 dpi	21 dpi	28 dpi
Fleet	drought-stressed	0.1±0.1	0.2±0.1	0.9±0.2ab	2.3±0.2a	3.4±0.2a	4.0±0.2a
	well-watered	0.0±0.0	0.4±0.2	1.0±0.2ab	2.1±0.2ab	2.7±0.3a	2.8±0.2b
	T	1.0	-0.9	-0.5	0.5	1.4*	4.0**
Franklin	drought-stressed	0.0	0.5±0.2	0.9±0.2ab	1.9±0.1ab	2.7±0.1a	4.3±0.2a
	well-watered	0.1±0.2	0.7±0.2	1.7±0.2a	1.6±0.1ab	1.9±0.2b	2.5±0.2b
	T	-1.5	-0.5	-2.7*	1.8	2.6*	6.1**
CSCRB8003	drought-stressed	0.0	0.1±0.1	0.3±0.1b	0.9±0.b	1.4±0.1c	2.3±0.2bc
	well-watered	0.0	0.4±0.1	0.7±0.1b	1.1±0.2b	1.3±0.2c	1.4±0.2c
	T	--	-1.7*	-2.3*	-0.4	0.2	4.5**
CSCRB8012	drought-stressed	0.0	0.2±0.1	0.5±0.1b	1.0±0.2b	1.9±0.2b	2.1±0.2bc
	well-watered	0.0	0.4±0.1	0.8±0.1ab	1.2±0.2b	1.3±0.2c	1.5±0.2c
	T	--	-1.2*	-2.0*	-0.7	2.6*	2.0*

T: independent sample test.

^a^ data in the table are mean±SE. Data followed by different lowercase letters are significantly different at *P*<0.05 level by Student-Newman-Keuls test.

*: *P*<0.05.

**: *P*<0.01.

**Fig 1:**
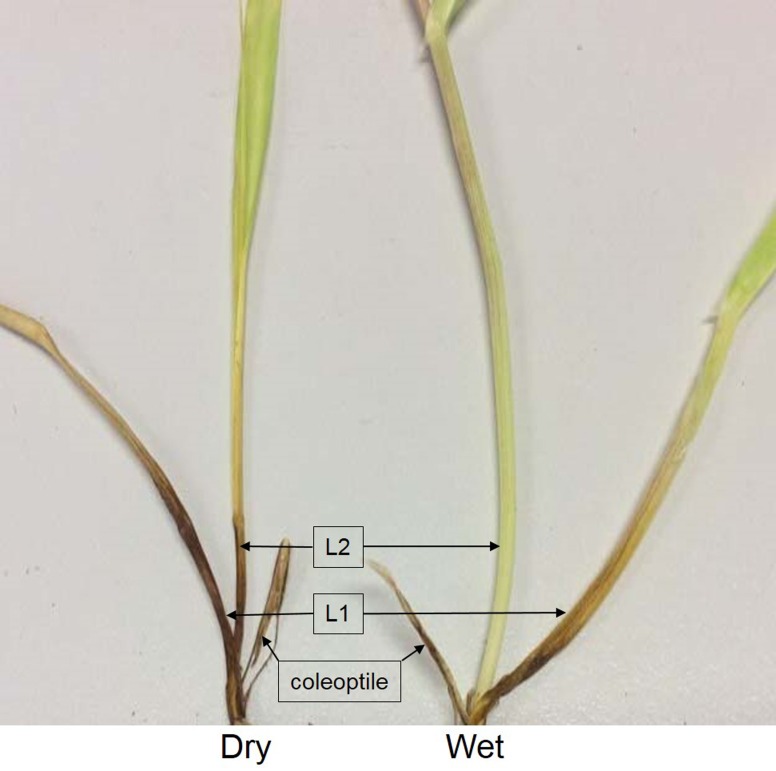
Showing typical difference in *Fusarium* crown rot severity between drought-stressed (left) and well-watered seedlings (right) of the susceptible barley genotype Fleet at 21 days post inoculation. L1: the first leaf sheath; L2: the second leaf sheath.

Following the initial infection phase, lesions developed rapidly in the stem base and the difference between the drought-stressed and well-watered seedlings became more obvious as the FCR infection progress. Compared with those well-watered seedlings, the drought-stressed seedlings of the two susceptible genotypes showed consistently higher disease ratings from 21 dpi. At this stage, the whole coleoptile and the bottom of L1 and L2 of the drought-stressed seedlings of Fleet and Franklin displayed widespread browning in epidermal cells (Fig 1). Similarly, FCR symptom of the drought-stressed seedlings of the two more resistant genotypes also became more severe compared to that of the well-watered seedlings toward the ends of the experiments, although the difference was not statistically significant.

### Water deficit affects the accumulation of fungal biomass

**Fig 2:**
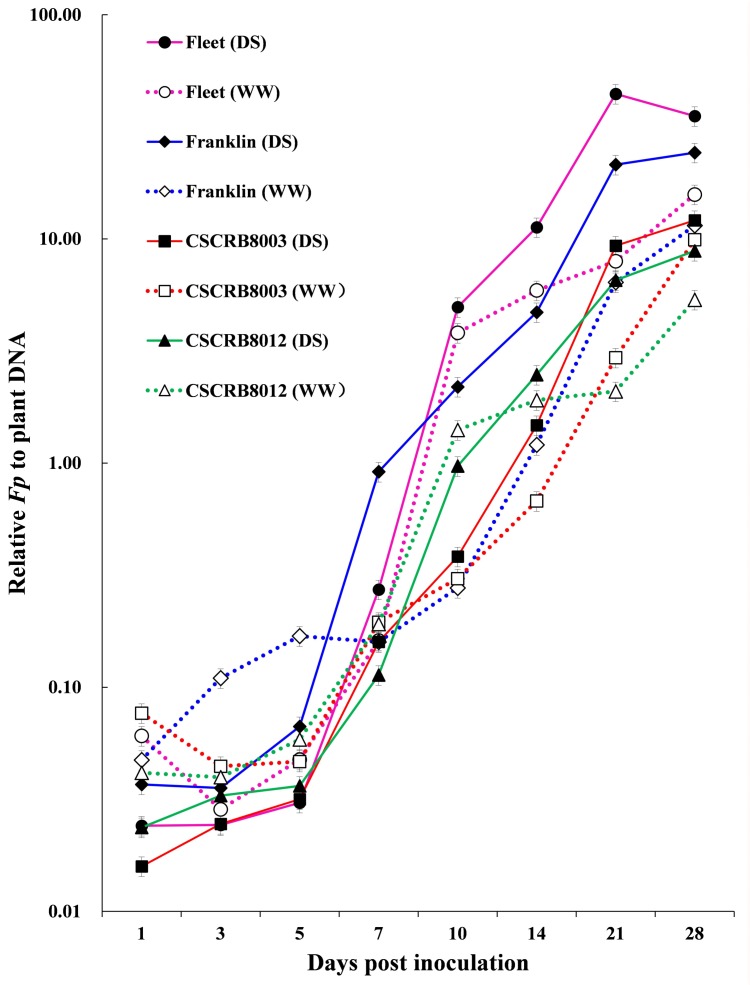
Relative *Fusarium pseudograminearum* biomass obtained by real-time quantitative PCR at different time points during *Fusarium* crown rot infection and development. DS: drought-stressed treatment; WW: well-watered treatment.

Three distinct phases of infection were identified by the relative fungal quantities measured at the eight time points of FCR disease development for all of the genotypes assessed (Fig 2 and S2 Table). Phase I stopped at 5 dpi during which infections of well-watered seedlings generally showed higher levels of relative fungal biomass than those drought-stressed oneswhile the increase of relative biomass in all seedlings was slow. Phase 2 started around 5 dpi and stopped at 21 dpi. This phase was characterized by a rapid increase in relative biomass. Significant differences among genotypes were observed during this phase. Larger quantities of fungal biomass in seedlings of Fleet and Franklin under drought-stressed condition were detected compared with that of the well-watered seedlings from 7 dpi (*t* = 2.32–8.95, *P*<0.05). Fungal biomass in seedlings of CSCRB8003 and CSCRB8012 under drought condition were also significantly higher compared to that of well-watered seedlings from 14 dpi (*t* = 0.68–6.37, *P*<0.05). Phase 3 covered the duration between 21 and 28 dpi, fungal biomass of the well-watered seedlings of all genotypes increased dramatically during this phase while that of drought-stressed seedlings increased more slowly (Fig 2). At 28 dpi, fungal biomass in the drought-stressed seedlings were significantly higher than that of well-watered ones (*t* = 3.05–10.22, *P*<0.01).

During the second and third phases of FCR infection, fungal biomass in the drought-stressed seedlings grew much faster than that in the well-watered ones. There was a strong correlation between disease rating and fungal biomass in both of the dry and wet treatments (*r* = 0.81; *P*<0.01).

### Microscopic analysis of fungal colonization of barley seedlings

To further investigate the difference in FCR infection between drought-stressed and well-watered seedlings histological analysis was conducted (Fig 2, Table 1 and S1 Table). Samples representing each of the three phases of FCR disease development were taken at 3, 7, and 28 dpi, respectively.

**Fig 3:**
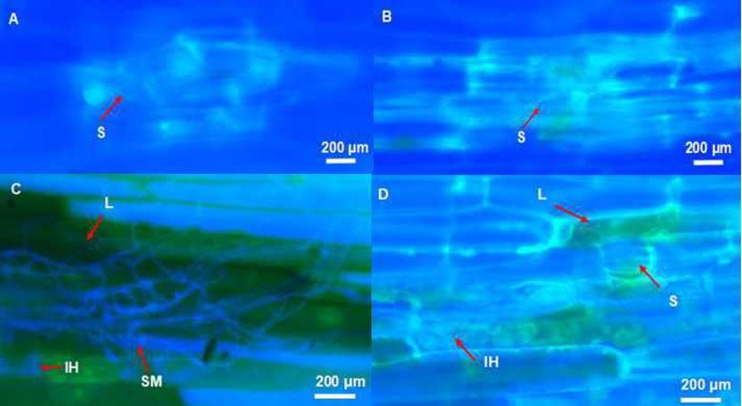
**The infection and colonization of barley coleoptile by *Fusarium pseudograminearum*** A: Small amounts of hyphae emerging from stomata and re-invading the surrounding epidermis cells at 3 dpi in the drought-stressed genotype Fleet. B: Intracellular invasive hyphae growing in epidermis cell adjacent to stomata at 3 dpi in the well-watered genotype Fleet. C: Surface mycelium growing through heavily infected tissue at 7 dpi in the drought-stressed genotype Franklin. D: Intracellular hyphae growing within epidermal cells at 7 dpi in the well-watered genotype Franklin. (Tissues were stained using Fluorescent brightener 28 and viewed under ultraviolet light.) S: stomata; L: lesion; IH: intracellular hyphae; SM: surface mycelium.

At 3 dpi, initial penetration of the epidermis of basal coleoptile occurred at a low level as thin hyphae and initial lesion (discoloration) formation were occasionally observed at stomata (Fig 3A and 3B). Most infection occurred at stomata and the adjacent epidermal cells. Small amounts of hyphae re-emerging from stomata and invading the surrounding epidermis cells on the abaxial coleoptile surface of seedling under drought condition were also observed (Fig 3A). In contrast, larger lesions in epidermis cell adjacent to stomata with intracellular invasive hyphae were observed in seedlings under well-watered condition (Fig 3B).

At 7 dpi, large quantities of mycelium on the exterior surface of infected tissue of seedlings under drought-stressed condition were detected (Fig 3C). Hyphae in the well-watered seedlings were predominantly observed to be within and across epidermal cells but not on the exterior surface (Fig 3D). Lesions in the coleoptile of Franklin and Fleet were darker and larger than those of CSCRB8003 and CSCRB8012 in both the drought-stressed and well-watered conditions. Although the basal tissues of L1 did not exhibit intra- or intercellular hyphae, the bases of trichome, epidermal cell and stoma displayed discoloration, indicating that penetration had occurred.

**Fig 4:**
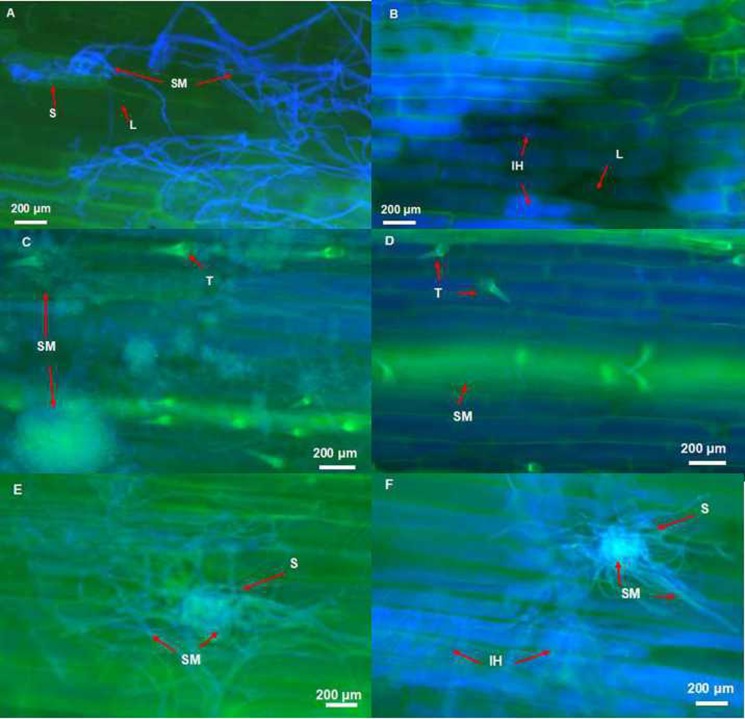
*Fusarium pseudograminearum* colonization of barley leaf sheath tissues. A: A mass of surface mycelium entering the open stoma at the base of heavily infected second leaf sheath at 28 dpi in the drought-stressed genotype Franklin. B: Intracellular hyphae growing within epidermal cells at the base of heavily infected second leaf sheath tissues at 28 dpi in the well-watered genotype Franklin. C: Large quantity of surface mycelium occurred around trichomes at 28 dpi in the first leaf sheath of the drought-stressed seedling of Fleet. D: A small amount of mycelium appeared around trichomes at 28 dpi in the first leaf sheath of the well-watered genotype Fleet. E: Large quantity of hyphae re-emerging from stoma at 28 dpi in the first leaf sheath of the drought-stressed genotype Fleet. F: Small quantity of hyphae re-emerging from stomata and a large quantity of intracellular hyphae growing within and across epidermal cells at 28 dpi in the first leaf sheath of the well-watered genotype Fleet. (Tissues were stained using Fluorescent brightener 28 and viewed under ultraviolet light.) S: stomata; L: lesion; IH: intracellular hyphae; SM: surface mycelium; T:trichome.

At 28 dpi, extensive *F*. *pseudograminearum* colonization was observed in both abaxial and adaxial surfaces of coleoptiles of all genotypes. At the same time point, bigger lesions and more dense hyphae were observed in leaf sheaths tissues of Fleet and Franklin than CSCRB8003 and CSCRB8012, and leaf sheaths in the drought-stressed seedlings typically showed more extensive hyphal growth compared to those in the well-watered condition (Fig 4A and 4B). Compared with those of the well-watered seedlings, denser surface mycelium network and pelotons were observed around trichomes and stoma at L1 and L2 of seedlings under drought-stress (Fig 4C and 4D, S1–S4 Figs).

Following the penetration, substomatal chambers are the major sites for hyphae accumulation (Figs 3A, 3B, 4E and 4F, S5–S8 Figs). After growing into the substomatal cavity, most of the invading hyphae re-emerged from stomata. It was observed frequently that such mycelium growing on the surface would invade surrounding epidermis cells again under drought-stressed conditions (Fig 4E, S5 and S7 Figs). However, under well-watered conditions, it seems that infection was mainly caused by invasive hyphae growing intracellularly although a small quantity of hyphae re-emerged from stomata was detected (Fig 4F, S6 and S8 Figs). Large quantities of surface mycelium wrapping around trichomes and coralliform were detected on abaxial surface of L1 in seedlings under drought-stress (Fig 4C, S1 and S3 Figs). Compared with those of the drought-stressed seedlings, less hyphae were observed in L1 epidermal cells in the well-watered ones (Fig 4D, S2 and S4 Figs). At the base of L2, a mass of hyphae on the cell surface entering the open stomata was observed under drought-stress (Fig 4A), while intensive lesions with intracellular hyphae were observed in only the well-watered seedling (Fig 4B).

### Effects of water deficit on trichome length and density

During the process of mycelium examination, difference in trichome length and density among the samples was observed. Significant difference in trichome length was detected between the drought-stressed and well-watered seedlings for each of the four barley genotypes assessed (Table 2, S3 Table, S9 and S10 Figs). Drought-stress significantly increased trichome length (by 12.4 to 88.0%; *P*<0.01) and trichome density (by 25.0 to 56.8%; *P*<0.01). Compared with the two resistant genotypes (CSCRB8012 and CSCRB8003), the two susceptible genotypes (Franklin and Fleet) have longer and denser trichomes under the drought-stressed condition. Trichome length and trichome density were positively related to fungal biomass of *F*. *pseudograminearum* (r = 0.53 and 0.58, respectively).

**Table 2:** Effects of drought on trichome traits measured on drought-stressed and well-watered seedlings at 14 days post inoculation.

Barley genotype	Length of trichome in μm ^a^				Number of trichome per square millimetre			
	drought-stressed	well-watered	Difference (%)	T	drought-stressed	well-watered	Difference (%)	T
Fleet	171.6±12.9a	91.3±3.7c	88.0	6.0**	4.3±0.1b	3.1±0.1a	38.7	9.0**
Franklin	156.5±4.9ab	92.3±4.2c	69.6	9.9**	5.8±0.2a	3.7±0.1a	56.8	9.1**
CSCRB8003	140.3±4.6b	124.8±3.1a	12.4	2.8*	2.0±0.1c	1.6±0.1b	25.0	1.6**
CSCRB8012	139.0±8.8b	112.2±5.6b	23.9	2.5*	2.0±0.1c	1.6±0.1b	25.0	2.9**

T: independent sample test.

^a^ data in the table are mean±SE. Data followed by different lowercase letters are significantly different at P<0.05 by the Student-Newman-Keuls test.

*: *P*<0.05.

**: *P*<0.01.

## Discussion

This is the first report on the direct relationship between pathogen quantity and FCR severity in FCR development under both drought-stressed and well-watered conditions in barley. Our data showed that the observed severity of FCR symptom reflected the quantity of pathogens in infected tissues of barley in both drought-stressed and well-watered conditions. During the mid- to late phases of FCR infection, fungal biomass of *F*. *pseudograminearum* and disease rating of FCR in the drought-stressed seedlings increased much faster than that in the well-watered seedlings. Results from this study also showed that drought-stress did not only affect the quantity of hyphae, but also the patterns of hyphae growth. Although intra- and inter-cellular hyphae were observed in both drought-stressed and well-watered seedlings, most of hyphae invading living plant cells were those intracellularly grown and few hyphae re-emerged from stomata in the well-watered seedling. In contrast, large quantities of hyphae re-emerged from stomata and invaded the surrounding epidermis cells via surface-grown mycelium in the drought-stressed seedling. The symptoms of FCR and the quantities of fungal biomass in the drought-stressed seedlings were significantly higher than those in the well-watered plants after 14 dpi, and this was especially the case in the two susceptible genotypes. It was also observed in this study that stomata are the main points of entry for *F*. *pseudograminearum* to infect coleoptile and leaf sheath tissues and that substomatal chambers are the major sites for accumulation of invading hyphae after colonization. These observations explain why initial lesions were mainly observed around stomata. Previous studies have repeatedly shown that stomata are important organ in pathogenic bacteria invasion [21, 22].

It is not difficult to understand why high moisture helps the initial infection of FCR as the *Fusarium* pathogens could need that condition to maintain vigor. However, it is not clear why water deficit enhanced the process of pathogen proliferation following the initial infection. This phenomenon is in contrast to that of *Fusarium* head blight (FHB) which can be caused by the same *Fusarium* isolates as those causing FCR [7, 10, 23]. It is intriguing that the *Fusarium* pathogens spread more quickly under drought if the infected tissue is at stem base (FCR) but the opposite is the case when the spike is infected [24]. Clearly, crown and spikes have different structures and they are also exposed to different microenvironments (temperature and humidity) due to their different distances from the soil surface. Previous studies showed that these factors affect the development of both FHB [25] and FCR [26].

It was also observed in this study that drought-stress increased the length and density of trichomes and that *Fusarium* hyphae grew around these structures. Compared with the two resistant genotypes, both of the susceptible genotypes have longer and denser trichomes and drought-stress further enhanced the difference. Trichome length and trichome density were positively related to fungal biomass of *F*. *pseudograminearum*. Further studies are required to clarify if the increased density and length of trichomes contributed to the difference in FCR severity between the well-watered and drought-stressed plants. However, trichomes are known to perform many biological functions including plant defense against insect predation [27] and adaptation of plants to drought-stressed environments [28].

## Supporting Information

S1 Fig Dense surface mycelium network and lesions occurred at abaxial surfaces of the first leaf sheath at 28 dpi in the drought-stressed seedling of Fleet. (Tissues were stained using Fluorescent brightener 28 and viewed under ultraviolet light.) L: lesion; IH: intracellular hyphae; SM: surface mycelium; T:trichome.
(TIF)

S2 Fig Intracellular hyphae and lesions occurred at abaxial surfaces of the first leaf sheath at 28 dpi in the well-watered seedling of Fleet. (Tissues were stained using Fluorescent brightener 28 and viewed under ultraviolet light.) L: lesion; IH: intracellular hyphae; T:trichome.
(TIF)

S3 Fig Large quantity of surface mycelium occurred around trichomes at 28 dpi in the first leaf sheath of the drought-stressed seedling of Fleet. (Tissues were stained using Fluorescent brightener 28 and viewed under ultraviolet light.) S: stomata; L: lesion; SM: surface mycelium; T:trichome.
(TIF)

S4 Fig A small amount of mycelium appeared around trichomes at 28 dpi in the first leaf sheath of the well-watered genotype Fleet. (Tissues were stained using Fluorescent brightener 28 and viewed under ultraviolet light.) L: lesion; SM: surface mycelium; T:trichome.
(TIF)

S5 Fig Large quantity of hyphae re-emerging from stoma at 28 dpi in the first leaf sheath of the drought-stressed genotype Fleet. (Tissues were stained using Fluorescent brightener 28 and viewed under blue light.) S: stomata; SM: surface mycelium.
(TIF)

S6 Fig Small quantity of hyphae re-emerging from stomata and a large quantity of intracellular hyphae growing within and across epidermal cells at 28 dpi in the first leaf sheath of the well-watered genotype Fleet. (Tissues were stained using Fluorescent brightener 28 and viewed under blue light.) S: stomata; IH: intracellular hyphae; SM: surface mycelium.
(TIF)

S7 Fig A great deal of hyphae re-emerging from stoma at 14 dpi in the coleoptile of the drought-stressed genotype CSCRB8003. (Tissues were stained using Fluorescent brightener 28 and viewed under ultraviolet light.) L: lesion; S: stomata; SM: surface mycelium.
(TIF)

S8 Fig Intracellular hyphae and lesions occurred around stomata at 14 dpi in the coleoptile of the well-watered genotype CSCRB8003. (Tissues were stained using Fluorescent brightener 28 and viewed under ultraviolet light.) L: lesion; S: stomata; IH: intracellular hyphae.
(TIF)

S9 Fig Longer and denser trichomes occurred at abaxial surfaces of the first leaf sheath at 14 dpi in the drought-stressed seedling of CSCRB8012. (Tissues were stained using Fluorescent brightener 28 and viewed under blue light.)
(TIF)

S10 Fig Less and shorter trichomes occurred at abaxial surfaces of the first leaf sheath at 14 dpi in the well-watered seedling of CSCRB8012. (Tissues were stained using Fluorescent brightener 28 and viewed under blue light.)
(TIF)

S1 Table Statistical results of FCR severity of barley genotypes assessed at different time points of postinoculation. (DOC)

S2 Table The mean values of threshold cycle of RT-qPCR of barley genotypes using *Actin* and *Tri5* genes. (DOC)

S3 Table The number and length of trichomes at the abaxial face of the first leaf sheath. The number and length of trichome at the abaxial face of the first leaf sheath measured at two different positions on each epidermal peel. Three microscope fields per peel and five peels for each barley genotype (non-inoculated controls) at 14 days postinoculation were examined. Trichomes lying over the veins were not considered in this study.
(DOC)
