# Supplementary figures and images for: Effects of Drought-Stress on Fusarium Crown Rot Development in Barley
Source: PLoS One. 2016 Dec 9;11(12):e0167304. doi: 10.1371/journal.pone.0167304 (PMC5147875; doi:10.1371/journal.pone.0167304)

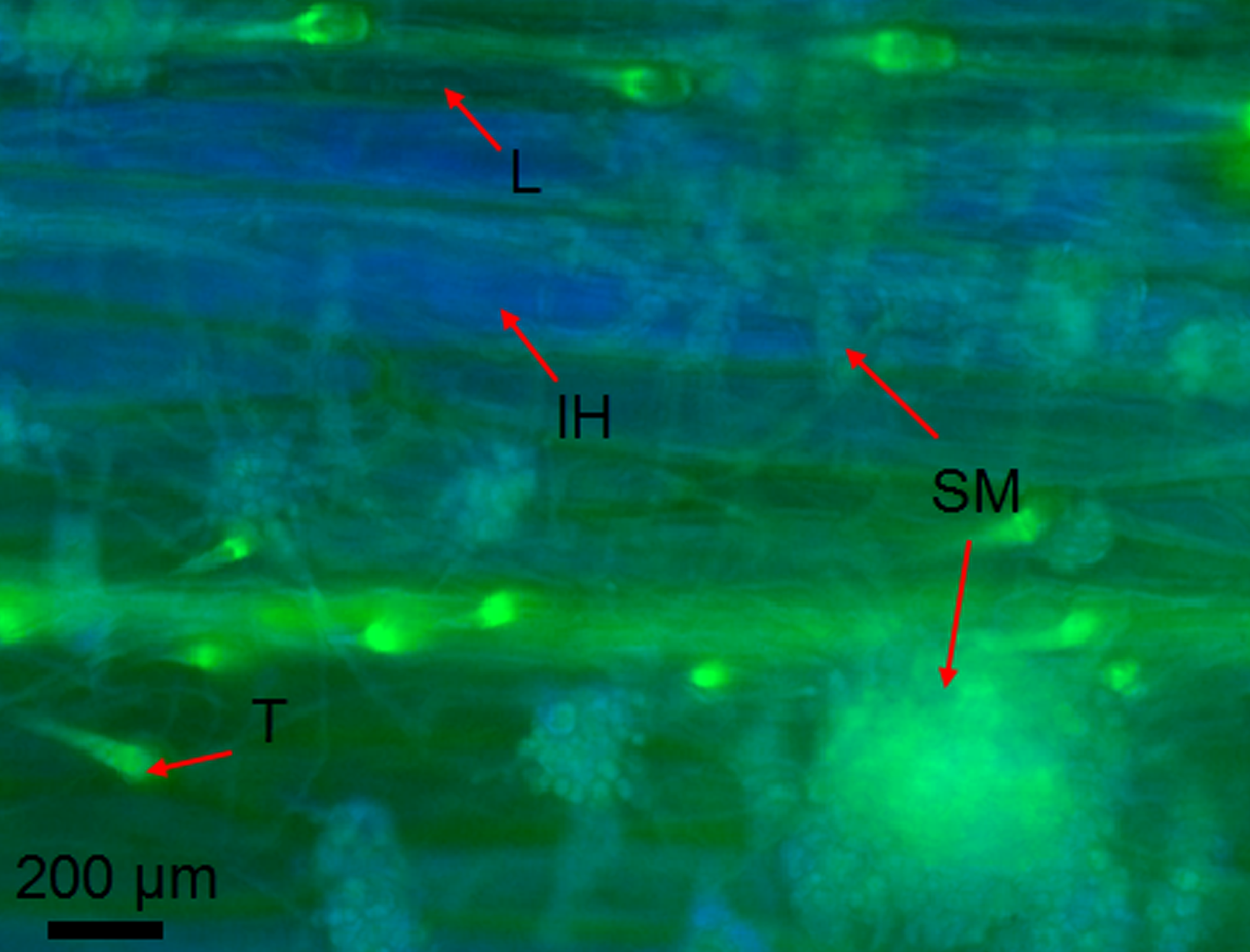

Supplement: S1 Fig — (Tissues were stained using Fluorescent brightener 28 and viewed under ultraviolet light.) L: lesion; IH: intracellular hyphae; SM: surface mycelium; T:trichome. (TIF) [file pone.0167304.s001.tif]

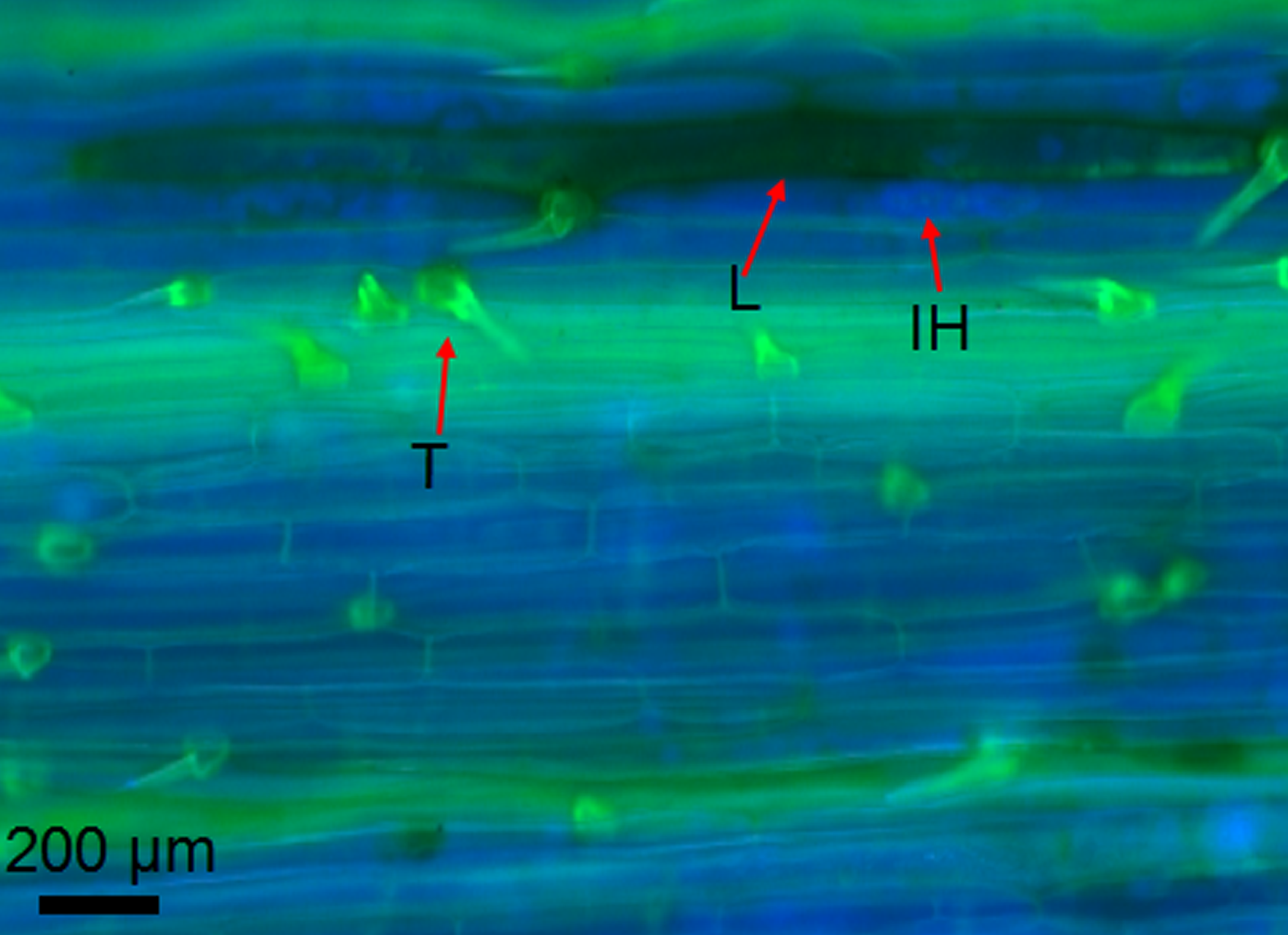

Supplement: S2 Fig — (Tissues were stained using Fluorescent brightener 28 and viewed under ultraviolet light.) L: lesion; IH: intracellular hyphae; T:trichome. (TIF) [file pone.0167304.s002.tif]

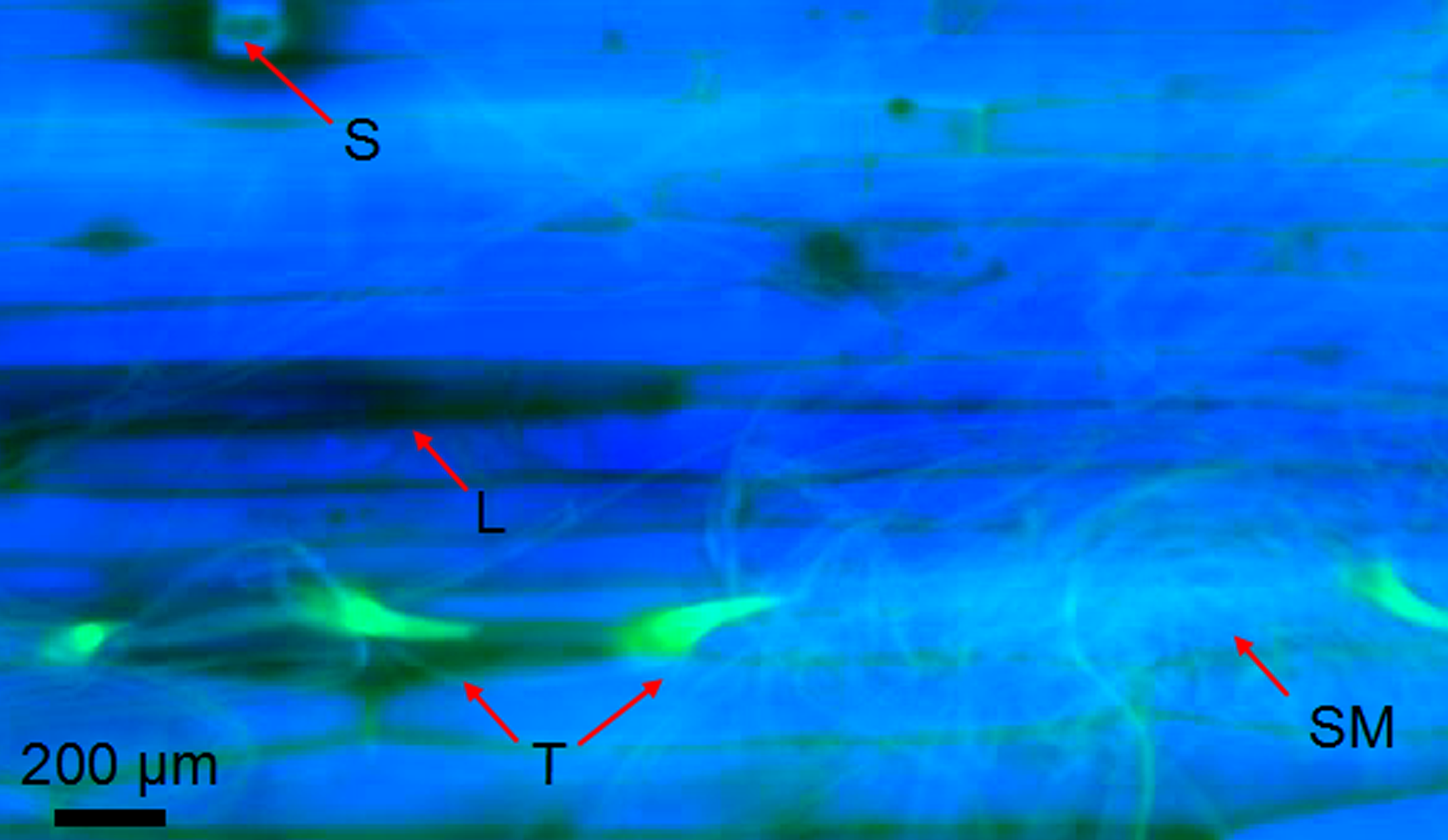

Supplement: S3 Fig — (Tissues were stained using Fluorescent brightener 28 and viewed under ultraviolet light.) S: stomata; L: lesion; SM: surface mycelium; T:trichome. (TIF) [file pone.0167304.s003.tif]

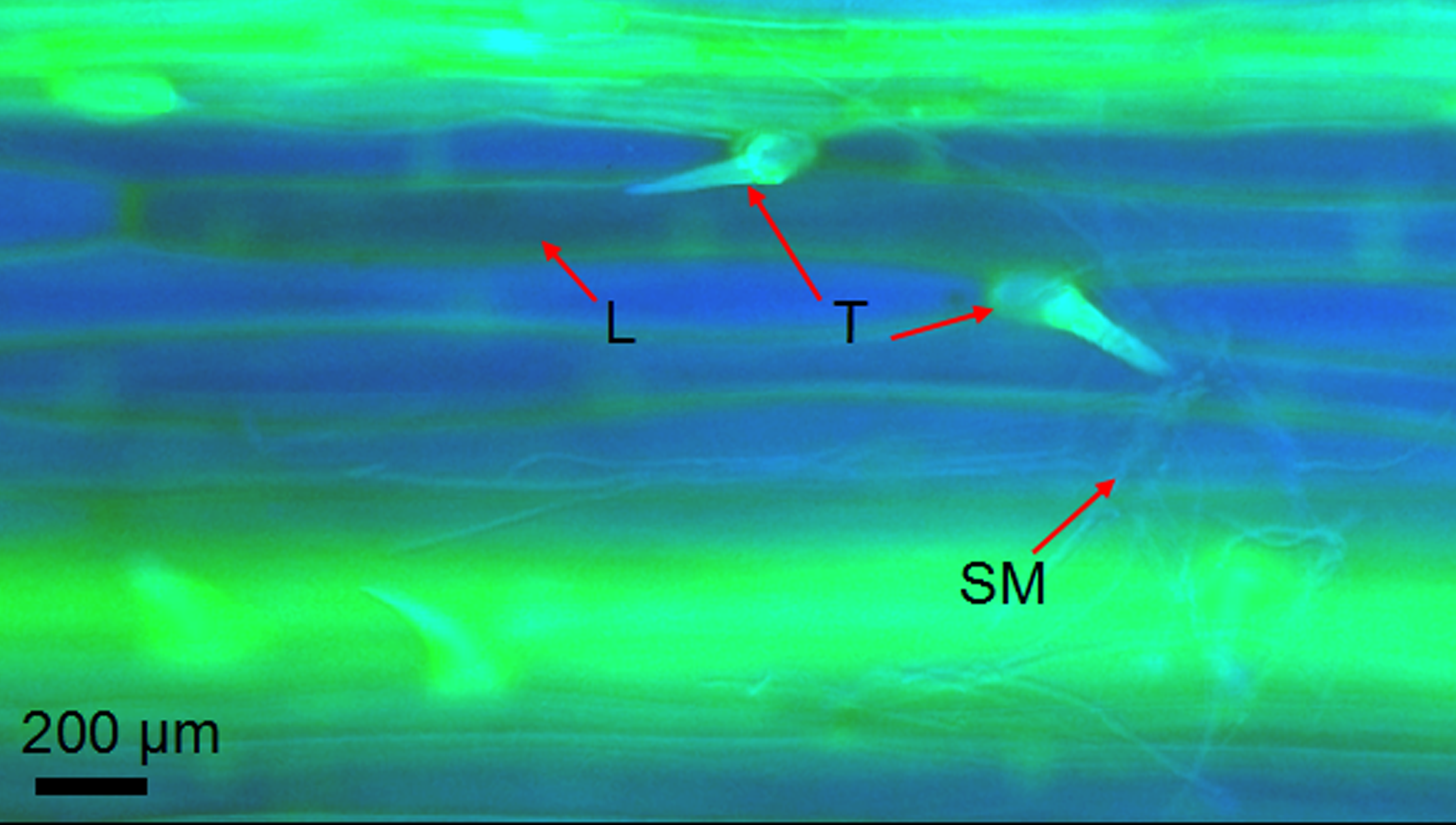

Supplement: S4 Fig — (Tissues were stained using Fluorescent brightener 28 and viewed under ultraviolet light.) L: lesion; SM: surface mycelium; T:trichome. (TIF) [file pone.0167304.s004.tif]

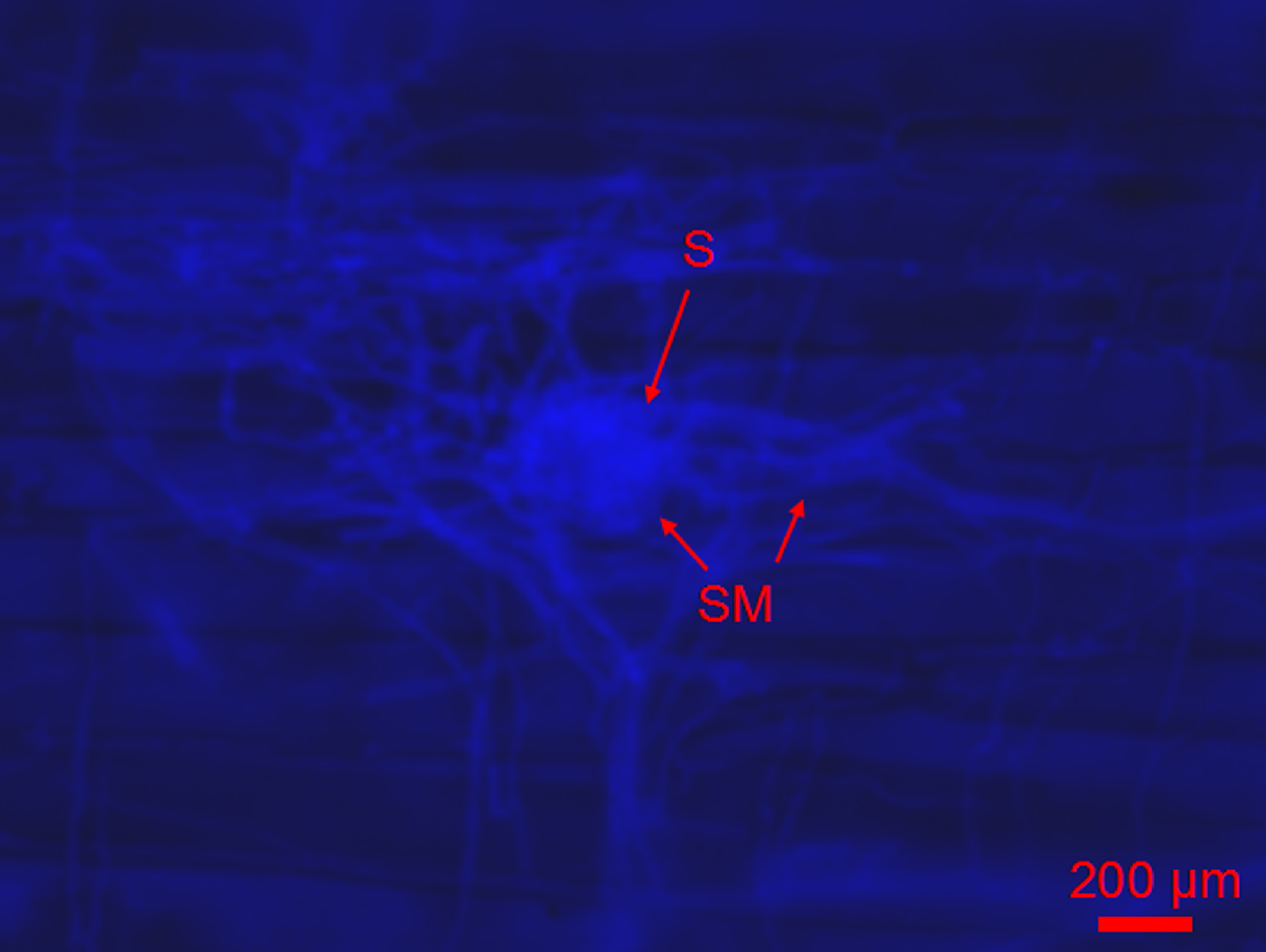

Supplement: S5 Fig — (Tissues were stained using Fluorescent brightener 28 and viewed under blue light.) S: stomata; SM: surface mycelium. (TIF) [file pone.0167304.s005.tif]

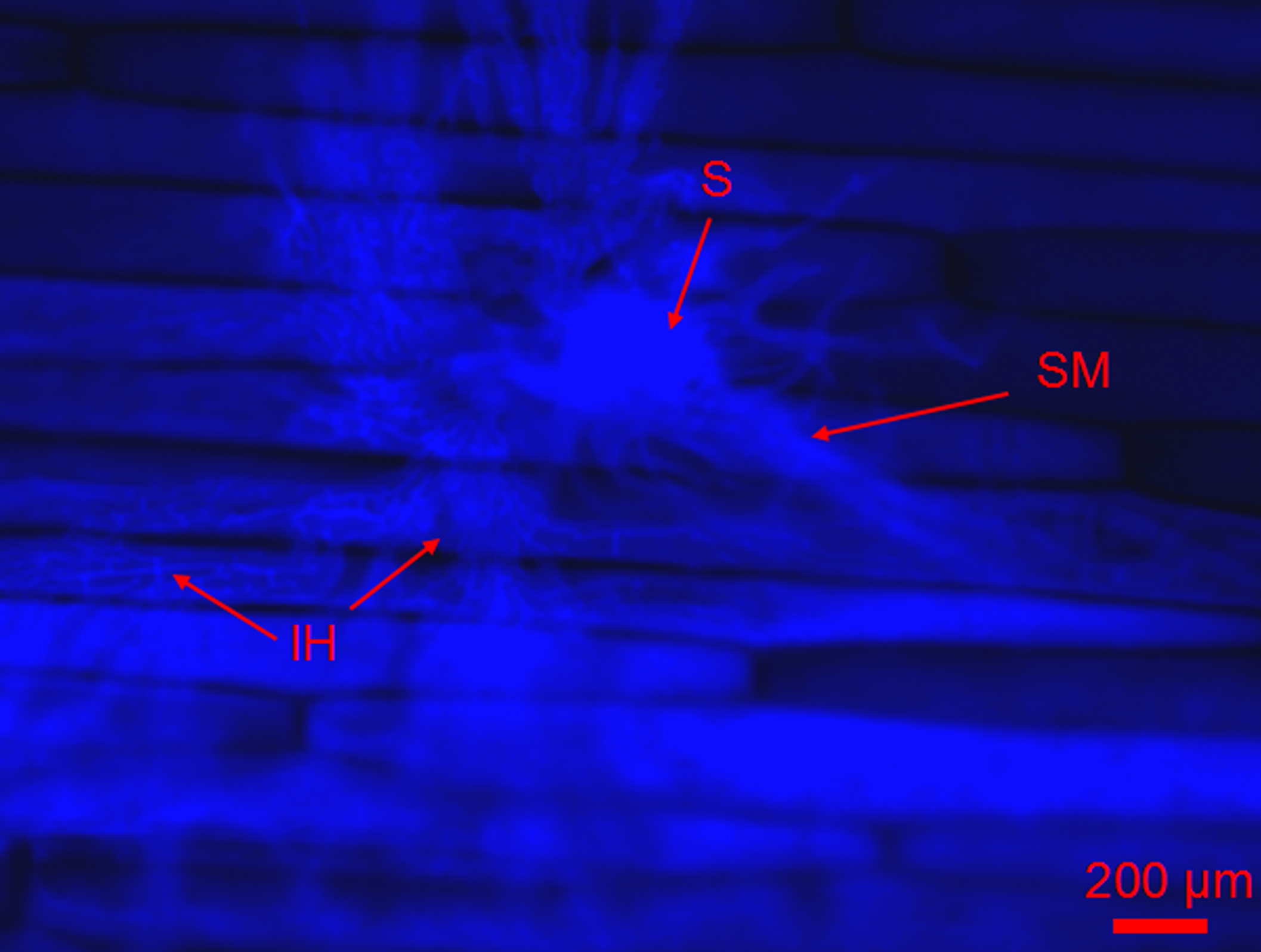

Supplement: S6 Fig — (Tissues were stained using Fluorescent brightener 28 and viewed under blue light.) S: stomata; IH: intracellular hyphae; SM: surface mycelium. (TIF) [file pone.0167304.s006.tif]

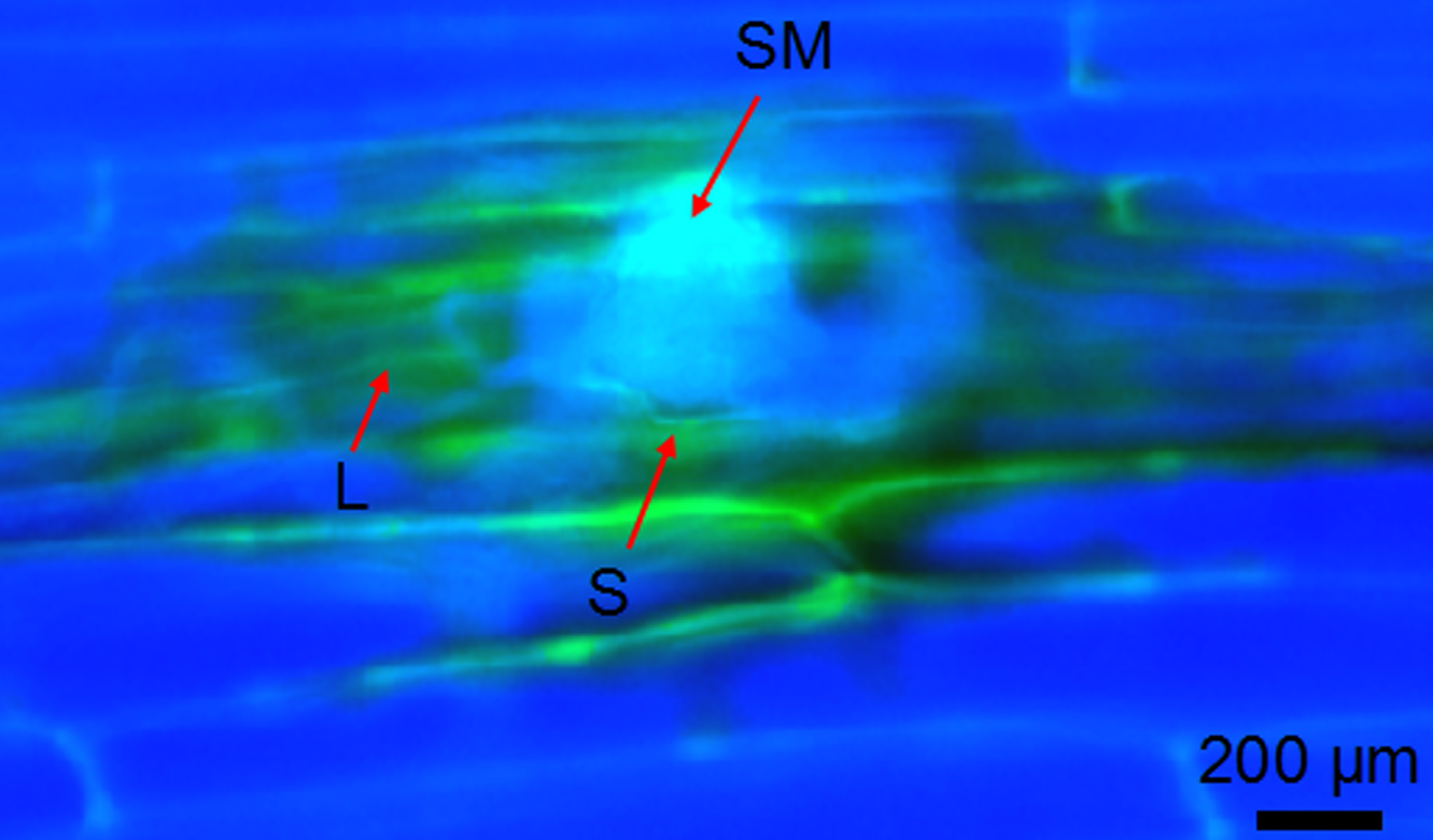

Supplement: S7 Fig — (Tissues were stained using Fluorescent brightener 28 and viewed under ultraviolet light.) L: lesion; S: stomata; SM: surface mycelium. (TIF) [file pone.0167304.s007.tif]

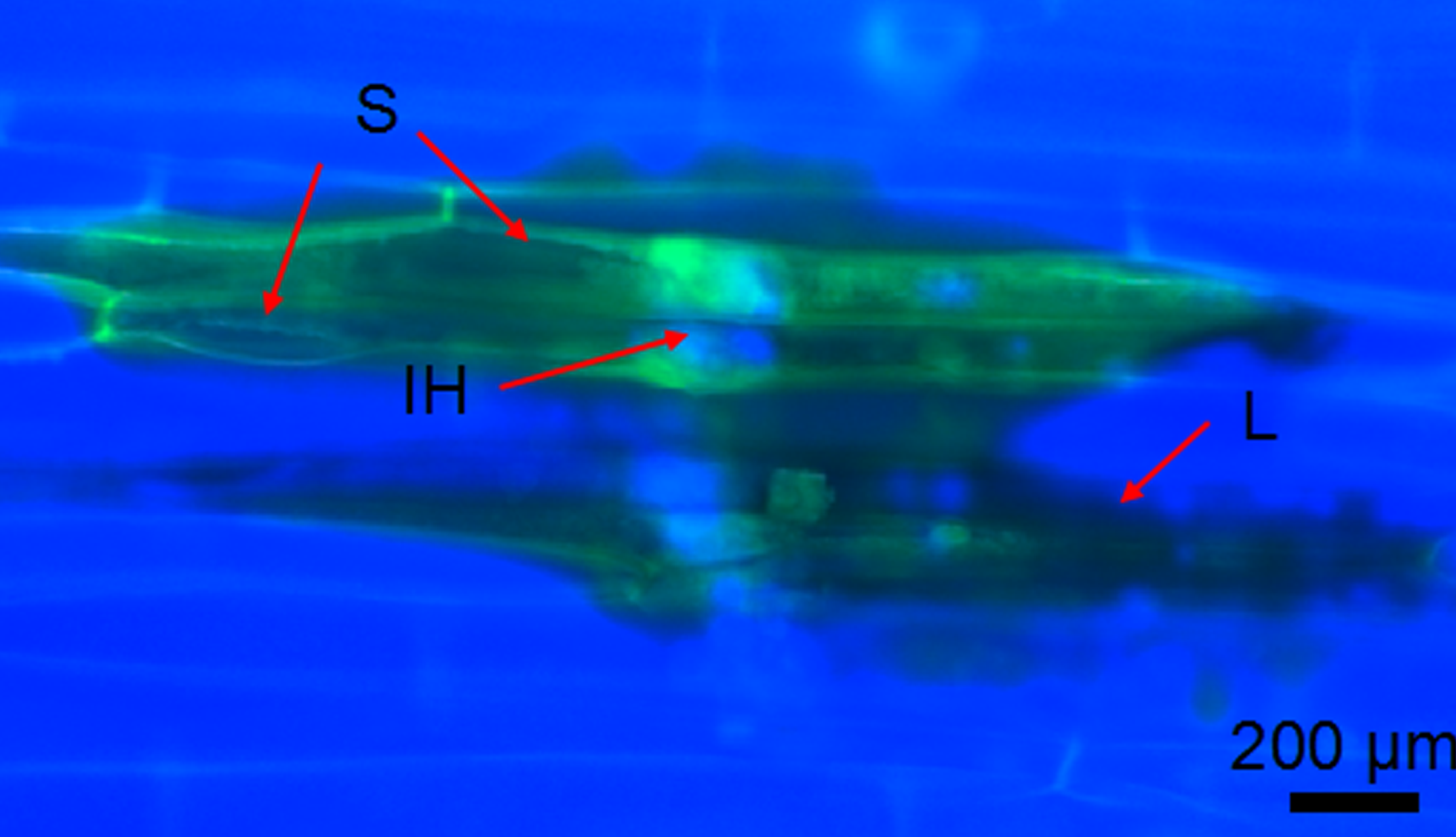

Supplement: S8 Fig — (Tissues were stained using Fluorescent brightener 28 and viewed under ultraviolet light.) L: lesion; S: stomata; IH: intracellular hyphae. (TIF) [file pone.0167304.s008.tif]

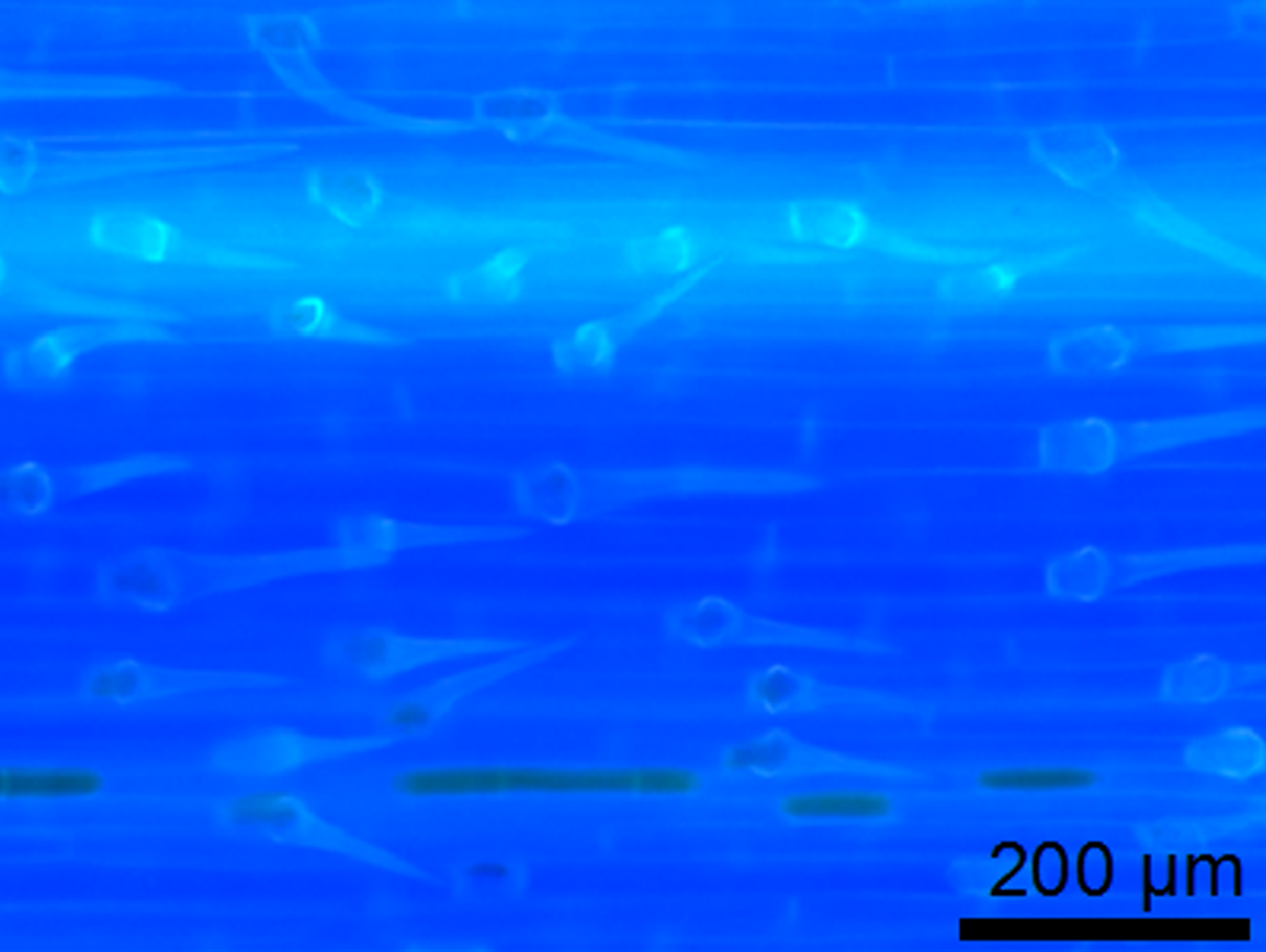

Supplement: S9 Fig — (Tissues were stained using Fluorescent brightener 28 and viewed under blue light.) (TIF) [file pone.0167304.s009.tif]

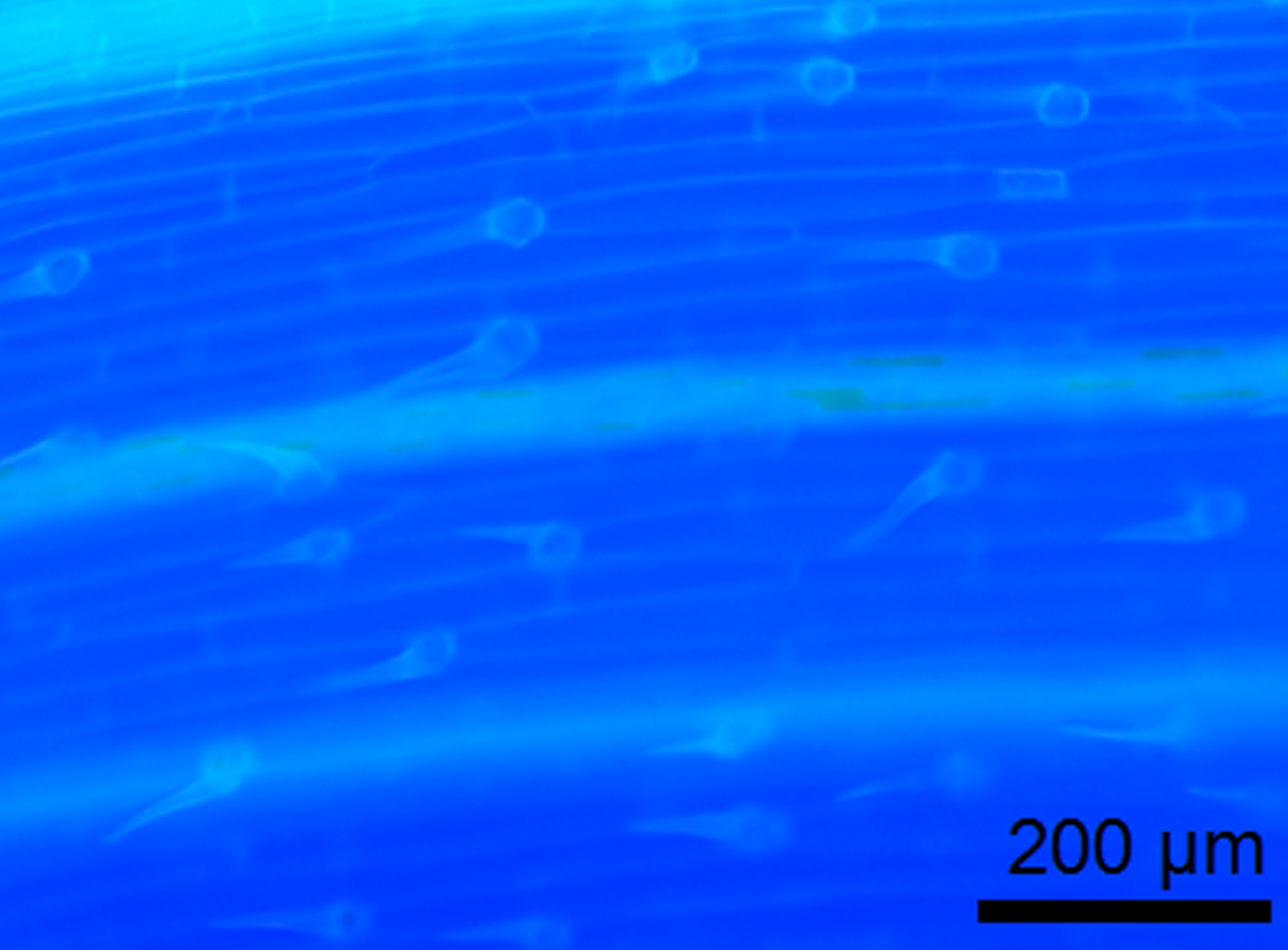

Supplement: S10 Fig — (Tissues were stained using Fluorescent brightener 28 and viewed under blue light.) (TIF) [file pone.0167304.s010.tif]
