# Supplementary material for: Effects of Drought-Stress on Fusarium Crown Rot Development in Barley
Source: PLoS One. 2016 Dec 9;11(12):e0167304. doi: 10.1371/journal.pone.0167304 (PMC5147875; doi:10.1371/journal.pone.0167304)
Supplement: S1 Table — (DOC) [file pone.0167304.s011.doc]

**S1 Table. Statistical results of FCR severity of barley genotypes assessed at different time points of postinoculation.**

| Barley genotype | Time * | Treatment | 0 | 1 | 2 | 3 | 4 | 5 |
| --- | --- | --- | --- | --- | --- | --- | --- | --- |
| Fleet | 5 dpi | drought-stressed | 14 | 1 |  |  |  |  |
|  |  | well-watered | 15 | 0 | 0 |  |  |  |
|  | 7 dpi | drought-stressed | 13 | 1 | 1 |  |  |  |
|  |  | well-watered | 10 | 4 | 1 |  |  |  |
|  | 10 dpi | drought-stressed | 6 | 5 | 4 |  |  |  |
|  |  | well-watered | 4 | 7 | 4 |  |  |  |
|  | 14 dpi | drought-stressed |  | 3 | 5 | 7 |  |  |
|  |  | well-watered |  | 2 | 9 | 4 |  |  |
|  | 21 dpi | drought-stressed |  |  | 2 | 5 | 8 |  |
|  |  | well-watered |  | 3 | 6 |  | 7 |  |
|  | 28 dpi | drought-stressed |  |  |  | 6 | 6 | 6 |
|  |  | well-watered |  | 2 | 5 | 7 | 5 |  |
|  |  |  |  |  |  |  |  |  |
| Franklin | 5 dpi | drought-stressed | 15 | 0 |  |  |  |  |
|  |  | well-watered | 14 | 1 |  |  |  |  |
|  | 7 dpi | drought-stressed | 8 | 6 | 1 |  |  |  |
|  |  | well-watered | 7 | 6 | 2 |  |  |  |
|  | 10 dpi | drought-stressed | 3 | 10 | 2 |  |  |  |
|  |  | well-watered | 1 | 3 | 11 |  |  |  |
|  | 14 dpi | drought-stressed |  | 3 | 11 | 1 |  |  |
|  |  | well-watered |  | 7 | 8 |  |  |  |
|  | 21 dpi | drought-stressed |  |  | 5 | 10 |  |  |
|  |  | well-watered |  | 4 | 8 | 3 |  |  |
|  | 28 dpi | drought-stressed |  |  |  | 3 | 6 | 9 |
|  |  | well-watered |  | 3 | 5 | 8 | 2 |  |
|  |  |  |  |  |  |  |  |  |
| CSCRB8003 | 5 dpi | drought-stressed | 15 |  |  |  |  |  |
|  |  | well-watered | 15 |  |  |  |  |  |
|  | 7 dpi | drought-stressed | 13 | 2 |  |  |  |  |
|  |  | well-watered | 9 | 6 |  |  |  |  |
|  | 10 dpi | drought-stressed | 10 | 5 |  |  |  |  |
|  |  | well-watered | 4 | 11 |  |  |  |  |
|  | 14 dpi | drought-stressed | 6 | 4 | 5 |  |  |  |
|  |  | well-watered | 5 | 4 | 6 |  |  |  |
|  | 21 dpi | drought-stressed |  | 9 | 6 |  |  |  |
|  |  | well-watered |  | 10 | 5 |  |  |  |
|  | 28 dpi | drought-stressed |  | 3 | 6 | 7 |  |  |
|  |  | well-watered |  | 12 | 7 |  |  |  |
|  |  |  |  |  |  |  |  |  |
| CSCRB8012 | 5 dpi | drought-stressed | 15 |  |  |  |  |  |
|  |  | well-watered | 15 |  |  |  |  |  |
|  | 7 dpi | drought-stressed | 12 | 3 |  |  |  |  |
|  |  | well-watered | 9 | 6 |  |  |  |  |
|  | 10 dpi | drought-stressed | 8 | 7 |  |  |  |  |
|  |  | well-watered | 3 | 12 |  |  |  |  |
|  | 14 dpi | drought-stressed | 4 | 7 | 4 |  |  |  |
|  |  | well-watered | 3 | 6 | 6 |  |  |  |
|  | 21 dpi | drought-stressed |  | 5 | 7 | 3 |  |  |
|  |  | well-watered |  | 11 | 4 |  |  |  |
|  | 28 dpi | drought-stressed |  | 5 | 4 | 6 |  |  |
|  |  | well-watered | 2 | 6 | 11 |  |  |  |

*dpi, days postinoculation
