# Supplementary material for: Effects of Drought-Stress on Fusarium Crown Rot Development in Barley
Source: PLoS One. 2016 Dec 9;11(12):e0167304. doi: 10.1371/journal.pone.0167304 (PMC5147875; doi:10.1371/journal.pone.0167304)
Supplement: S2 Table — (DOC) [file pone.0167304.s012.doc]

**S2 Table. The mean values of threshold cycle of RT-qPCR of barley genotypes using *Actin* and *Tri5* genes.**

| Time * | Treatment | Fleet | | Franklin | | CSCRB8003 | | CSCRB8012 | |
| --- | --- | --- | --- | --- | --- | --- | --- | --- | --- |
|  |  | *Tri5* | *Actin* | *Tri5* | *Actin* | *Tri5* | *Actin* | *Tri5* | *Actin* |
|  | CK | 29.023 | 25.662 | 29.149 | 26.065 | 29.506 | 29.456 | 29.264 | 25.612 |
| 1 dpi | drought-stressed | 34.374 | 25.892 | 33.684 | 28.230 | 30.502 | 29.022 | 30.529 | 26.905 |
|  | well-watered | 33.112 | 26.589 | 34.060 | 28.203 | 30.830 | 27.365 | 32.101 | 29.040 |
| 3 dpi | drought-stressed | 34.087 | 25.635 | 32.977 | 27.501 | 31.052 | 28.535 | 29.656 | 27.314 |
|  | well-watered | 33.907 | 26.365 | 32.302 | 29.243 | 31.471 | 28.812 | 34.524 | 25.390 |
| 5 dpi | drought-stressed | 35.329 | 25.586 | 32.210 | 27.591 | 30.863 | 24.933 | 32.212 | 26.980 |
|  | well-watered | 32.167 | 25.359 | 29.721 | 27.420 | 30.514 | 26.091 | 33.669 | 26.153 |
| 7 dpi | drought-stressed | 30.042 | 25.604 | 29.886 | 28.783 | 29.753 | 28.353 | 31.354 | 26.979 |
|  | well-watered | 30.620 | 25.473 | 31.363 | 27.913 | 26.977 | 26.543 | 31.574 | 27.878 |
| 10 dpi | drought-stressed | 26.615 | 26.105 | 28.827 | 28.906 | 31.023 | 29.141 | 28.547 | 27.088 |
|  | well-watered | 27.359 | 26.475 | 29.969 | 26.429 | 30.976 | 27.912 | 28.020 | 27.587 |
| 14 dpi | drought-stressed | 26.222 | 26.802 | 28.077 | 28.463 | 29.276 | 29.226 | 27.566 | 28.148 |
|  | well-watered | 27.117 | 26.813 | 28.462 | 27.773 | 28.660 | 27.613 | 29.152 | 28.560 |
| 21 dpi | drought-stressed | 24.994 | 28.536 | 27.874 | 30.606 | 26.166 | 30.267 | 26.921 | 28.651 |
|  | well-watered | 27.420 | 28.396 | 29.518 | 28.945 | 27.654 | 29.091 | 31.249 | 30.457 |
| 28 dpi | drought-stressed | 27.443 | 29.801 | 27.049 | 30.346 | 27.002 | 29.796 | 27.170 | 28.324 |
|  | well-watered | 27.531 | 27.605 | 28.528 | 28.656 | 27.752 | 30.258 | 27.198 | 29.520 |

*dpi, days postinoculation
