# Supplementary material for: Effects of Drought-Stress on Fusarium Crown Rot Development in Barley
Source: PLoS One. 2016 Dec 9;11(12):e0167304. doi: 10.1371/journal.pone.0167304 (PMC5147875; doi:10.1371/journal.pone.0167304)
Supplement: S3 Table — The number and length of trichome at the abaxial face of the first leaf sheath measured at two different positions on each epidermal peel. Three microscope fields per peel and five peels for each barley genotype (non-inoculated controls) at 14 days postinoculation were examined. Trichomes lying over the veins were not considered in this study. (DOC) [file pone.0167304.s013.doc]

**S3 Table. The number and length of trichome at the abaxial face of the first leaf sheath.**

| Barley genotype | Treatment | Number of trichome per 5 square millimetre | | | | | | | | | | | | | | |
| --- | --- | --- | --- | --- | --- | --- | --- | --- | --- | --- | --- | --- | --- | --- | --- | --- |
| Fleet | drought-stressed | 25 | 21 | 20 | 17 | 21 | 19 | 22 | 20 | 23 | 20 | 23 | 21 | 26 | 22 | 23 |
|  | well-watered | 16 | 14 | 16 | 15 | 17 | 12 | 16 | 14 | 16 | 17 | 16 | 15 | 16 | 14 | 16 |
| Franklin | drought-stressed | 27 | 26 | 25 | 26 | 25 | 35 | 30 | 32 | 27 | 35 | 30 | 32 | 29 | 27 | 27 |
|  | well-watered | 24 | 18 | 17 | 21 | 18 | 20 | 22 | 23 | 16 | 19 | 15 | 17 | 16 | 18 | 16 |
| CSCRB8003 | drought-stressed | 9 | 10 | 10 | 13 | 9 | 8 | 11 | 12 | 9 | 11 | 10 | 12 | 9 | 7 | 10 |
|  | well-watered | 6 | 9 | 5 | 13 | 6 | 7 | 7 | 12 | 6 | 10 | 5 | 11 | 6 | 8 | 9 |
| CSCRB8012 | drought-stressed | 11 | 8 | 9 | 7 | 10 | 8 | 13 | 9 | 7 | 10 | 11 | 13 | 11 | 12 | 12 |
|  | well-watered | 10 | 12 | 9 | 9 | 7 | 9 | 7 | 10 | 6 | 7 | 7 | 8 | 7 | 7 | 6 |
|  |  | Length of trichome in μm | | | | | | | | | | | | | | |
| Fleet | drought-stressed | 163 | 212 | 99 | 119 | 112 | 226 | 125 | 204 | 86 | 193 | 195 | 183 | 209 | 234 | 214 |
|  | well-watered | 78 | 103 | 75 | 70 | 80 | 97 | 107 | 116 | 97 | 78 | 97 | 106 | 94 | 75 | 97 |
| Franklin | drought-stressed | 142 | 190 | 135 | 133 | 175 | 179 | 171 | 144 | 149 | 153 | 143 | 165 | 139 | 145 | 184 |
|  | well-watered | 96 | 77 | 78 | 91 | 95 | 93 | 92 | 92 | 74 | 100 | 81 | 87 | 73 | 130 | 123 |
| CSCRB8003 | drought-stressed | 160 | 142 | 123 | 142 | 160 | 131 | 116 | 155 | 165 | 133 | 108 | 129 | 129 | 163 | 148 |
|  | well-watered | 112 | 130 | 134 | 136 | 133 | 112 | 116 | 143 | 132 | 121 | 140 | 125 | 116 | 120 | 102 |
| CSCRB8012 | drought-stressed | 190 | 180 | 158 | 133 | 128 | 95 | 88 | 116 | 121 | 195 | 169 | 138 | 109 | 157 | 108 |
|  | well-watered | 136 | 130 | 132 | 116 | 133 | 129 | 82 | 132 | 87 | 89 | 113 | 87 | 82 | 102 | 133 |

The number and length of trichome at the abaxial face of the first leaf sheath measured at two different positions on each epidermal peel. Three microscope fields per peel and five peels for each barley genotype (non-inoculated controls) at 14 days postinoculation were examined. Trichomes lying over the veins were not considered in this study.
